# Supplementary material for: Validation of the Italian version of the Dark Tetrad at Work scale
Source: PLoS One. 2024 Feb 23;19(2):e0298880. doi: 10.1371/journal.pone.0298880 (PMC10889854; doi:10.1371/journal.pone.0298880)
Supplement: S1 Table — (DOCX) [file pone.0298880.s001.docx]

**S1**

Items, descriptive statistics, and factor loadings of the four-factor model of the DTW scale (Italian version).

| Items | M (SD) | Skewness | Kurtosis | Factor loadings |
| --- | --- | --- | --- | --- |
| *Narcisismo (Narcissism)* |  |  |  |  |
| La mia posizione lavorativa è prestigiosa | 2.61 (0.99) | 0.28 | -0.29 | 0.53 |
| Valgo molto di più dei miei colleghi | 1.80 (0.94) | 1.04 | 0.38 | 0.87 |
| Esigo rispetto al lavoro | 3.80 (1.05) | -0.71 | -0.08 | 0.43 |
| Le persone mi prestano sempre attenzione al lavoro | 3.18 (0.91) | -0.13 | -0.30 | 0.47 |
| Gli altri mi ammirano al lavoro | 2.91 (0.98) | -0.01 | -0.43 | 0.60 |
| Mi piace essere al centro dell'attenzione al lavoro | 2.05 (1.09) | 0.91 | 0.13 | 0.74 |
| *Machiavellismo (Machiavellianism)* |  |  |  |  |
| Non mi fido degli altri al lavoro | 2.40 (1.08) | 0.44 | -0.49 | 0.73 |
| Al lavoro, devi sempre pensare solo a te stesso | 2.05 (1.04) | 0.80 | -0.07 | 0.82 |
| Al lavoro, le persone si pugnalano alle spalle per fare carriera | 2.75 (1.21) | 0.17 | -0.87 | 0.65 |
| Al lavoro, le persone sono motivate solo dal guadagno personale | 3.03 (1.09) | -0.07 | -0.68 | 0.68 |
| *Psicopatia (Psychopathy)* |  |  |  |  |
| Non mi interessa se il mio comportamento al lavoro ferisce gli altri | 1.60 (0.87) | 1.61 | 2.40 | 0.73 |
| Mi è stato detto che agisco avventatamente al lavoro | 1.58 (0.95) | 1.84 | 3.01 | 0.64 |
| Quando sono al lavoro, tendo a non pensare alle conseguenze delle mie azioni | 1.47 (0.79) | 1.92 | 4.01 | 0.71 |
| Mi piace scroccare ai miei colleghi | 1.45 (0.78) | 1.85 | 3.19 | 0.66 |
| Sono piuttosto insensibile quando sono al lavoro | 1.66 (0.92) | 1.51 | 1.94 | 0.76 |
| Non mi importa se accidentalmente ferisco qualcuno al lavoro | 1.34 (0.68) | 2.43 | 7.06 | 0.88 |
| *Sadismo (Sadism)* |  |  |  |  |
| Mi piace guardare il mio capo che urla contro i miei colleghi | 1.23 (0.66) | 3.59 | 14.20 | 0.77 |
| Posso dominare gli altri al lavoro usando la paura | 1.16 (0.47) | 4.03 | 21.50 | 0.82 |
| È divertente guardare le persone commettere errori al lavoro | 1.30 (0.71) | 2.79 | 8.19 | 0.78 |
| Non mi stanco mai di prendere in giro i miei colleghi | 1.37 (0.75) | 2.34 | 5.77 | 0.69 |
| Riderei se vedessi qualcuno essere licenziato | 1.11 (0.48) | 5.24 | 30.40 | 1.01 |
| Faccio sogni ad occhi aperti in cui faccio male alle persone con cui lavoro | 1.13 (0.48) | 4.73 | 26.20 | 0.75 |
