# Supplementary material for: Validation of the Italian version of the Dark Tetrad at Work scale
Source: PLoS One. 2024 Feb 23;19(2):e0298880. doi: 10.1371/journal.pone.0298880 (PMC10889854; doi:10.1371/journal.pone.0298880)
Supplement: S2 Table — (DOCX) [file pone.0298880.s002.docx]

**S2**

Descriptive statistics and scores distribution of the DTW scales in the Italian sample (N = 300).

|  | Narcissism | Machiavellianism | Psychopathy | Sadism |
| --- | --- | --- | --- | --- |
| Mean | 2.72 | 2.55 | 1.51 | 1.21 |
| Median | 2.67 | 2.50 | 1.33 | 1.00 |
| Range | 1-4.67 | 1-5 | 1-4.33 | 1-4.33 |
| Standard Deviation | 0.67 | 0.87 | 0.59 | 0.42 |
| Skewness | 0.35 | 0.32 | 1.71 | 3.68 |
| Kurtosis | 0.30 | -0.26 | 3.69 | 18.6 |
| Percentiles |  |  |  |  |
| *20* | 2.17 | 1.75 | 1.00 | 1.00 |
| *50* | 2.67 | 2.50 | 1.33 | 1.00 |
| *80* | 3.17 | 3.25 | 2.00 | 1.33 |
| *90* | 3.50 | 3.75 | 2.33 | 1.67 |
| *95* | 3.83 | 4.00 | 2.72 | 2.00 |
