# Supplementary material for: Validation of the Italian version of the Dark Tetrad at Work scale
Source: PLoS One. 2024 Feb 23;19(2):e0298880. doi: 10.1371/journal.pone.0298880 (PMC10889854; doi:10.1371/journal.pone.0298880)
Supplement: Attachment — Submitted filename: Response to Reviewers.docx [file pone.0298880.s004.docx]

Dear Dr Ramos-Villagrasa,

We would like to thank you for your positive evaluation of our manuscript and for the additional comments, which we have implemented in the manuscript. Below, we provide a point-by-point response (in **bold**) explaining how we have addressed each of your comments.

We look forward to the outcome of your assessment.

Best regards,

The authors

1) In the abstract, you mention the reliability coefficient for three out of the four dimensions of the Dark Tetrad. It would be preferable to include all four for a more comprehensive presentation.

**You are right, it made little sense reporting only three out of four reliability coefficients. Therefore, we have updated the abstract as follows: “*The Italian DTW scale demonstrated acceptable internal consistency, with reliability coefficients of ω = .77 for narcissism, ω = .80 for Machiavellianism, and ω = .81 for both psychopathy and sadism.*”**

2) In both the abstract and the limitations section, I believe the results related to Narcissism should be approached with caution. Since everything has been measured using self-report instruments, there is a possibility that socially desirable associations may be influencing the outcomes, as indicated by previous research cited in your article.

**Thank you for this comment. We have updated both the abstract (“*In contrast, narcissism exhibited a unique pattern: It correlated positively with positive workplace behaviors and negatively with counterproductive behaviors toward the organization, but it was also found to be a significant predictor of workplace bullying. This finding may reflect multidimensional nature of narcissism, but a note of caution is warranted in interpreting this result, as all measurements relied on self-report instruments, introducing the possibility of socially desirable associations influencing the outcomes.*”) and the limitations section (Page 17: “*Finally, it is essential to acknowledge that employing self-report data introduces potential sources of bias, including social desirability bias and common method bias. Consequently, caution is advised when interpreting the findings, particularly those related to narcissism, as individuals scoring high on this trait are known to exhibit heightened sensitivity to social desirability concerns [22; 48].*”) to acknowledge that these results should be approached with caution. We have also added an additional reference [48] concerning the influence of social desirability in narcissism:**

**48. Kowalski CM, Rogoza R, Vernon PA, Schermer JA. The Dark Triad and the self-presentation variables of socially desirable responding and self-monitoring. Personality and Individual Differences. 2018;120:234-237.**

3) On page 8, there is no need to mask the university's name.

**The university’s name is now unmasked (Page 8: “*The study was approved by the Ethics Committee of the University of Trieste, Italy, and was conducted in accordance with the Helsinki Declaration.*”).**
